# Bibliometric analysis of research trends and emerging insights of osteoarthritis and chondrocyte hypertrophy

**DOI:** 10.3389/fsurg.2025.1538339

**Published:** 2025-04-10

**Authors:** Jiajin Fang, Shuai Wang

**Affiliations:** ^1^ School of Clinical Medicine, Weifang Medical University, Weifang, Shandong, China; ^2^ Department of Sports Medical and Rehabilitation, Peking University Shenzhen Hospital, Shenzhen, Guangdong, China; ^3^ Pain Management Department, Zhejiang Provincial Hospital of Traditional Chinese Medicine, Hangzhou, China

**Keywords:** osteoarthritis, chondrocyte hypertrophy, bibliometric, hotspots, WoSCC

## Abstract

**Background:**

This study aims to systematically analyze the intersection of OA and chondrocyte hypertrophy using bibliometric methods, providing an quantitative and comprehensive overview of the current research status and emerging trends in this field.

**Methods:**

Relevant publications were retrieved from the Web of Science Core Collection database using the search query TS = (“chondrocyte* hypertroph*” OR “hypertrophic chondrocyte*” OR “cartilage hypertroph*”) AND (“osteoarthriti*” OR “OA” OR “degenerative arthritis”). Several bibliometric tools, including Vosviewer, CiteSpace, the R package (bibliometrix), and Excel 2021, were utilized to systematically analyze the publications on the intersection of chondrocyte hypertrophy and OA.

**Results:**

A total of 639 publications, published between 1995 and 2025, were identified. The findings indicate a steady global increase in research on OA and chondrocyte hypertrophy, with an increasing number of studies being published in high-impact journals, suggesting a promising developmental trajectory. China and the United States are leading in this field. OSTEOARTHRITIS AND CARTILAGE is identified as the core journal in this area, while ANNALS OF THE RHEUMATIC DISEASES has the highest impact factor among the top publishing journals. Keyword analysis reveals that research hotspots primarily focus on stem cells, tissue engineering, cartilage repair, inflammation, oxidative stress, autophagy, apoptosis, senescence, and related bioactive factors.

**Conclusion:**

This study elucidates the current research status and trends at the intersection of OA and chondrocyte hypertrophy, providing crucial references for future research. Future studies should continue to focus on these potential therapeutic approaches, key phenotypes, and regulatory mechanisms, enhance international cooperation to develop more effective strategies and treatments for OA.

## 1 Introduction

Osteoarthritis (OA) is the most common joint disease, with its prevalence continuously rising due to the aging global population (1). This condition significantly impacts patients' quality of life and imposes a substantial burden on public health systems (2). The most critical pathological changes in OA involve the degeneration and wear of articular cartilage, with chondrocytes playing a pivotal role in this process (3). However, current OA treatment strategies primarily focus on symptom management rather than addressing the underlying pathological causes. Most pharmacological treatments aim at pain relief and inflammation control (4). A deeper understanding of the chondrocyte phenotype during OA pathogenesis can provide new insights into the disease mechanism and propose potential therapeutic approaches (5).

During the pathological progression of OA, chondrocytes undergo a series of stress responses, including changes in biomechanical status, disruption of the extracellular matrix, increased expression of inflammatory factors, and alterations in metabolic states (6). These changes collectively lead to modifications in the phenotype and function of chondrocytes, including hypertrophic-like changes characterized by increased catabolism and inhibited anabolism (7). As the disease progresses, the cartilage extracellular matrix gradually degrades, further exposing and hydrolyzing type II collagen in chondrocytes, creating a vicious cycle that accelerates chondrocyte hypertrophy and exacerbates the secretion and release of pro-inflammatory factors, worsening joint cartilage damage (8, 9).

A notable feature of chondrocyte hypertrophy is the terminal differentiation of chondrocytes. Terminally differentiated chondrocytes exhibit characteristics similar to those of growth plate chondrocytes (10). This not only disrupts the existing cartilage matrix but also leads to the formation of abnormal mineral deposits, further aggravating structural damage to the joints. In the advanced stages of OA, the degree of chondrocyte hypertrophy becomes more pronounced, with cartilage tissue showing manifestations of an osteochondral complex, severely impacting joint function (11).

An increasing number of researchers are focusing on this area, leading to numerous new discoveries in cell and molecular biology as well as tissue engineering. Multiple signaling pathways are implicated in chondrocyte hypertrophy during (IHH, BMP, Wnt, etc) OA, and bioengineering technologies such as 3D printing and stem cell modulation can inhibit hypertrophic differentiation of chondrocytes, offering multidimensional therapeutic strategies for OA (12). These advancements are filling existing knowledge gaps. A better understanding of the mechanisms related to the chondrocytes hypertrophy during the pathological process of OA, and the exploration of potential therapeutic methods, hold profound significance and promising prospects (13).

Bibliometrics is a discipline that applies quantitative methods to the statistical analysis, evaluation, and assessment of scholarly publications. It plays a crucial role in evaluating scientific research, analyzing disciplinary development trends, assessing academic influence, facilitating knowledge dissemination and exchange, supporting research management and decision-making (14). Since the 21st century, advancements in information and computer technology have continuously expanded the research methods and application fields of bibliometrics. The application of big data analysis, data mining, and visualization techniques enables bibliometrics to handle large-scale datasets and conduct more in-depth analyses (15).

In this study, we utilized various bibliometric software tools, including Vosviewer, CiteSpace, the R package (bibliometrix), and Excel 2021, to systematically analyze publications in the intersecting fields of chondrocyte hypertrophy and OA. We aimed to provide a systematic analysis of the current research status and development trends in this intersecting fields. This will not only help the academic community understand the research dynamics in this area but also provide important references and guidance for related research.

## 2 Method

### 2.1 Literature search and data collection

**Figure 1:**
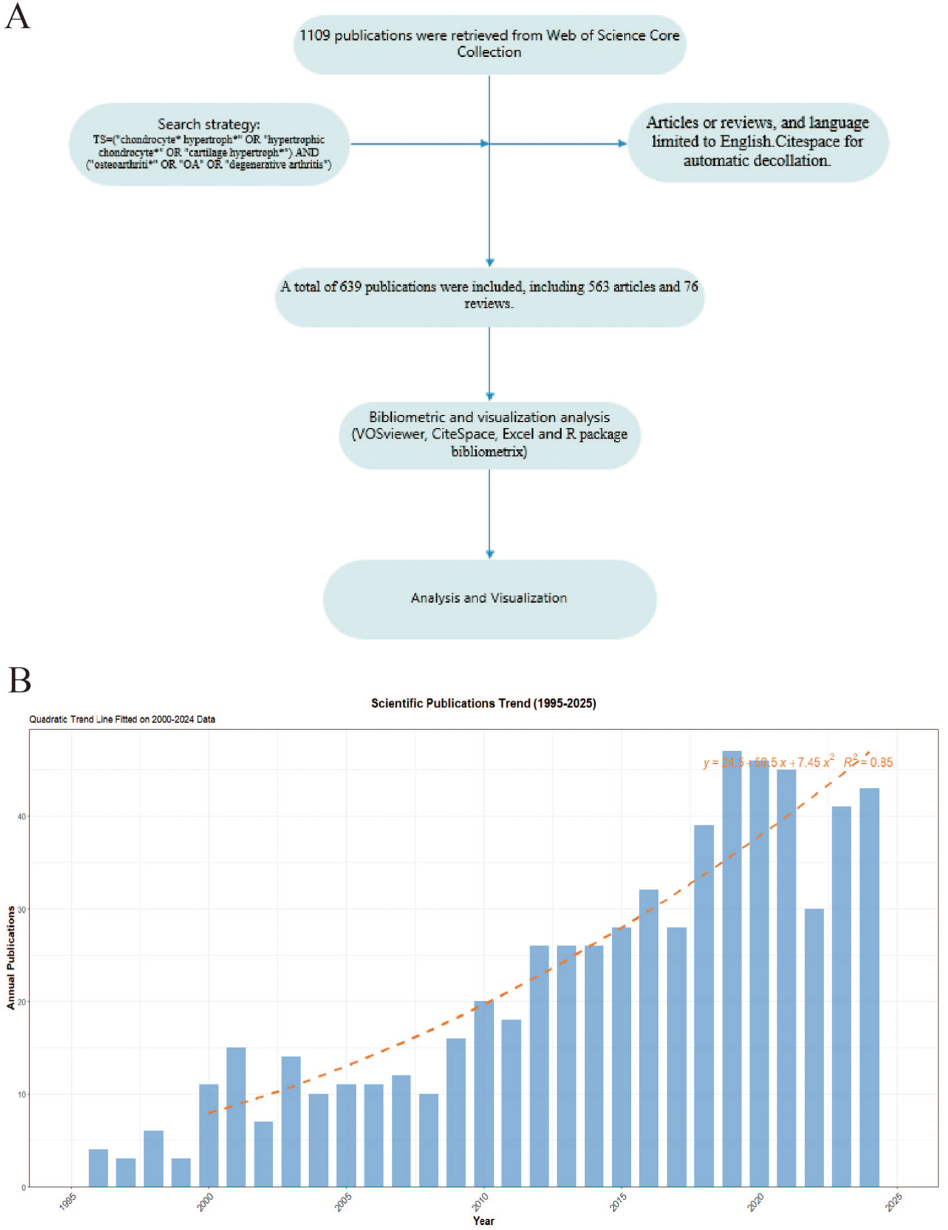
**(A)** The flowchart of this study. **(B)** The annual publication number of OA and Chondrocyte Hypertrophy from 1995–2025.

Web of Science Core Collection (WOSCC) is one of the most influential scientific citation databases globally, containing millions of records from numerous academic journals, conference papers, and books (16). The literatures included in WOSCC undergo rigorous peer review and screening, ensuring data quality and reliability, thus providing a solid foundation for bibliometric analysis. On March 20, 2025, we conducted a literature search in WOSCC using the search query TS = (“chondrocyte* hypertroph*” OR “hypertrophic chondrocyte*” OR “cartilage hypertroph*”) AND (“osteoarthriti*” OR “OA” OR “degenerative arthritis”), with publication type restricted to articles or reviews, and language limited to English. We selected the “Full Record and Cited References” option for subsequent bibliometric analysis. We used Citespace for automatic decollation. Figure 1A showed the flowchart of this study.

### 2.2 Data analysis and visualization

We utilized several bibliometric software tools, including the R package Bibliometrix, VOSviewer 1.6.20, Microsoft Office Excel 2021, and CiteSpace 6.2.R6, to analyze the bibliometric characteristics of the included publications and to generate various charts and visualizations. The analysis included keyword co-occurrence, citation and co-citation networks, as well as country, institution, and author collaboration networks. We also identified high-impact publications, journals, countries, institutions, and authors. Through these results, we aim to explore the research hotspots, frontiers, influence, and collaboration patterns in the intersecting fields of OA and chondrocyte hypertrophy. All network analyses in VOSviewer employed the association strength normalization method with clustering resolution set to 1.0 and minimum cluster size of 5. For CiteSpace burst detection, parameters were configured with time slicing (1-year intervals), node types (keywords/institutions/countries), g-index (*k* = 25), and burst detection gamma = 0.8 to optimize sensitivity. These parameter settings align with software defaults recommended by VOSviewer and CiteSpace developers. Adhering to these defaults helps minimize subjective intervention while ensuring methodological transparency and reproducibility.

## 3 Results

### 3.1 Publication summary

Based on the aforementioned search strategy, we extracted a total of 639 publications spanning from 1995–2025, comprising 563 articles and 76 reviews. These publications were authored by 3,468 researchers from 722 institutions across 45 countries and were published in 243 different journals. The first publication in this field appeared in 1995, this study revealed that the expression of type X collagen in osteoarthritic cartilage (enhanced in calcified zones and peri-clonal regions) indicates chondrocyte reversion to a hypertrophic phenotype, driving pathological matrix remodeling and mineralization imbalance (17). The annual number of publications in this field remained below ten until 2000. However, since 2010, there has been a steady increase in publication volume, peaking in 2019 with 47 publications. We created a growth prediction model for the number of published papers based on Excel 2021: *y* = 24.5 + 58.5*x* + 7.45*x*^2^, *R*^2^ = 0.85, The quadratic regression model demonstrated strong predictive validity, with an *R*^2^ of 0.85 indicating that 85% of the variance in publication trends is explained by the model (Figure 1B).

### 3.2 Analysis of countries

**Figure 2:**
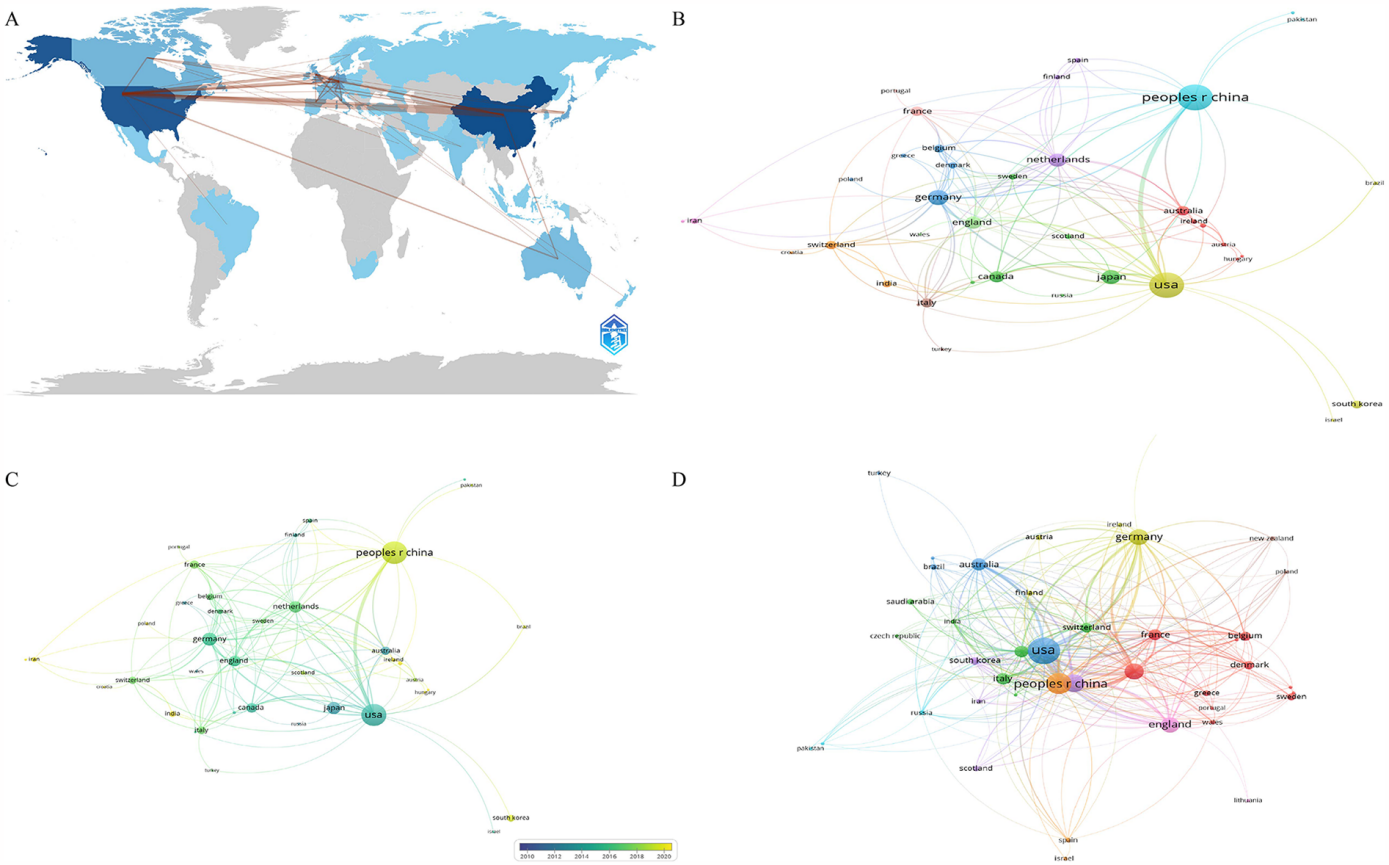
The analysis of countries. **(A)** Collaboration map of countries, shades of the color represented the publication number, the thickness of the line represented the frequency of cooperation. **(B,C)** The network map of countries, the size of the nodes represented the publication number **(B)**. The color from blue to yellow represented the average appearing year **(C)**. **(D)** The citation analysis of countries, the size of the nodes represented the total citation number.

Table 1 presents the top ten countries in terms of publication volume. China leads with the highest number of publications (*N* = 187), followed by the United States (*N* = 172), with no other country exceeding 50 publications. Apart from China, all the top ten publishing countries are developed nations. In terms of Total Citation (TC), the United States has the highest count (TC = 11,156), followed by China (TC = 5,075). The United States and China form the top tier in this field, significantly leading over other countries. Figure 2A shows the Country Collaboration Map, where the color intensity represents the number of publications, and the depth of the connecting lines represents the collaboration between countries. Figures 2B,C presents the co-authorship analysis among countries. Figure 2D shows the citation analysis of countries.

**Table 1:** The information of high contribution countries.

Country	Publications	Total Citations
China	187	5,075
USA	172	11,156
Germany	75	4,640
Japan	71	4,595
United Kingdom	53	2,991
Canada	49	2,129
Netherland	41	2,038
Australia	32	2,110
France	30	1,031
South Korea	22	647

### 3.3 Analysis of high-impact journals and publications

**Figure 3:**
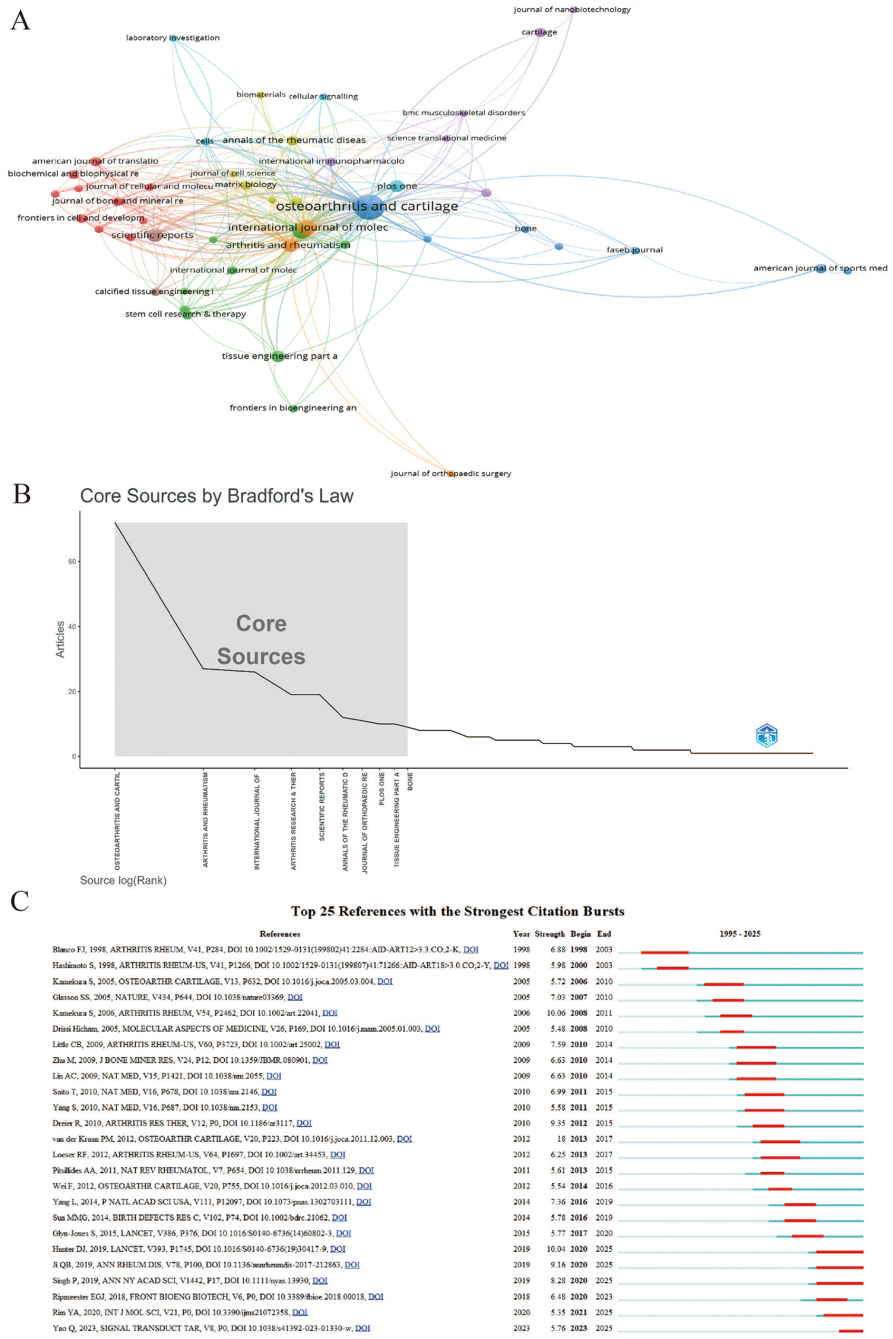
The analysis of journals and publications. **(A)** The network map of journals' citations, the size of the nodes represented the citation number. **(B)** Core journals according to the Bradford's Law. **(C)** Top 25 references with strongest citation burst.

Table 2 displays the top ten journals by publication volume. OSTEOARTHRITIS AND CARTILAGE is the most central journal in this field (*N* = 72, TC = 3,652, H-index = 32), holding an absolutely leading position. According to Bradford's Law, journals with more than seven publications are considered as core journals in this field (18). Among the top ten journals by publication volume, the one with the highest impact factor is ANNALS OF THE RHEUMATIC DISEASES (IF = 27.4), indicating that research in this field has gained considerable attention and can be published in high-impact journals. Figure 3A shows the journal citation network analysis, and Figure 3B illustrates the core journals in this field according to Bradford's Law. Table 3 presents the most cited documents. The most cited one investigated the critical role of matrix metalloproteinase MMP-9 in skeletal growth plate development, revealing its regulatory mechanisms in angiogenesis and hypertrophic chondrocyte apoptosis (19). The second one reviewed molecular mechanism with pathological progression, emphasizing the central role of chondrocyte phenotypic transition in OA, thereby providing a theoretical foundation for developing novel therapeutic strategies targeting hypertrophic pathways (20). The third most-cited study demonstrated that MMP-13-deficient mice exhibit significant resistance to cartilage erosion in OA models, while chondrocyte hypertrophy and osteophyte formation remain unaffected (21). The fourth explored the interplay between angiogenesis and inflammation in OA, with implications for the regulatory mechanisms underlying chondrocyte hypertrophy (22). The fifth revealed that MMP-13-deficient mice display skeletal developmental abnormalities during endochondral ossification, particularly affecting terminal differentiation of chondrocytes in the growth plate and subsequent bone replacement processes (23).

**Table 2:** The information of high impact journals.

Journals	N	Total Citations	H-index	IF
Osteoarthritis and Cartilage	72	3,652	32	7
Arthritis and rheumatism	27	4,046	27	8.23
Arthritis research & therapy	19	979	17	4.4
International journal of molecular sciences	26	926	15	4.9
Scientific reports	19	555	13	4.6
Annals of the rheumatic diseases	12	880	12	27.4
Journal of biological chemistry	8	327	8	4.0
Journal of bone and mineral research	8	405	8	5.1
Tissue engineering part A	10	311	8	3.5
Biochemical and biophysical research communications	8	186	7	2.5

**Table 3:** The top 10 most cited publications.

Title	Year	Journals	Total citations
MMP-9/gelatinase B is a key regulator of growth plate angiogenesis and apoptosis of hypertrophic chondrocytes	2001	Journal of Cell Biology	533
Chondrocyte hypertrophy and osteoarthritis: role in initiation and progression of cartilage degeneration?	2011	Osteoarthritis and Cartilage	525
Matrix metalloproteinase 13-deficient mice are resistant to osteoarthritic cartilage erosion but not chondrocyte hypertrophy or osteophyte development	2009	Arthritis & Rheumatism	508
Osteoarthritis, angiogenesis and inflammation	2005	Rheumatology	508
Altered endochondral bone development in matrix metalloproteinase 13-deficient mice	2004	Development	498
Emerging regulators of the inflammatory process in osteoarthritis	2014	Nature Reviews Rheumatology	489
Critical roles for collagenase-3 (MMP-13) in postnatal growth plate development	2004	Proceedings of the National Academy of Sciences (PNAS)	461
Osteoarthritis development in novel experimental mouse models induced by knee joint instability	2005	Osteoarthritis and Cartilage	451
Transcriptional regulation of endochondral ossification by HIF-2*α* during skeletal growth and osteoarthritis development	2010	Nature Medicine	431
Cartilage-like gene expression in differentiated human stem cell spheroids: a comparison of bone marrow-derived and adipose tissue-derived stromal cells	2003	Arthritis & Rheumatism	388

Highly-cited studies have collectively demonstrated the pivotal role of chondrocyte hypertrophy in OA pathogenesis, revealing multi-layered regulatory mechanisms spanning molecular drivers, signaling cascades, and clinically validated biomarkers. Additionally, we performed an analysis of the top 25 citation bursts, as shown in Figure 3C.

### 3.4 Analysis of institutions and authors

**Figure 4:**
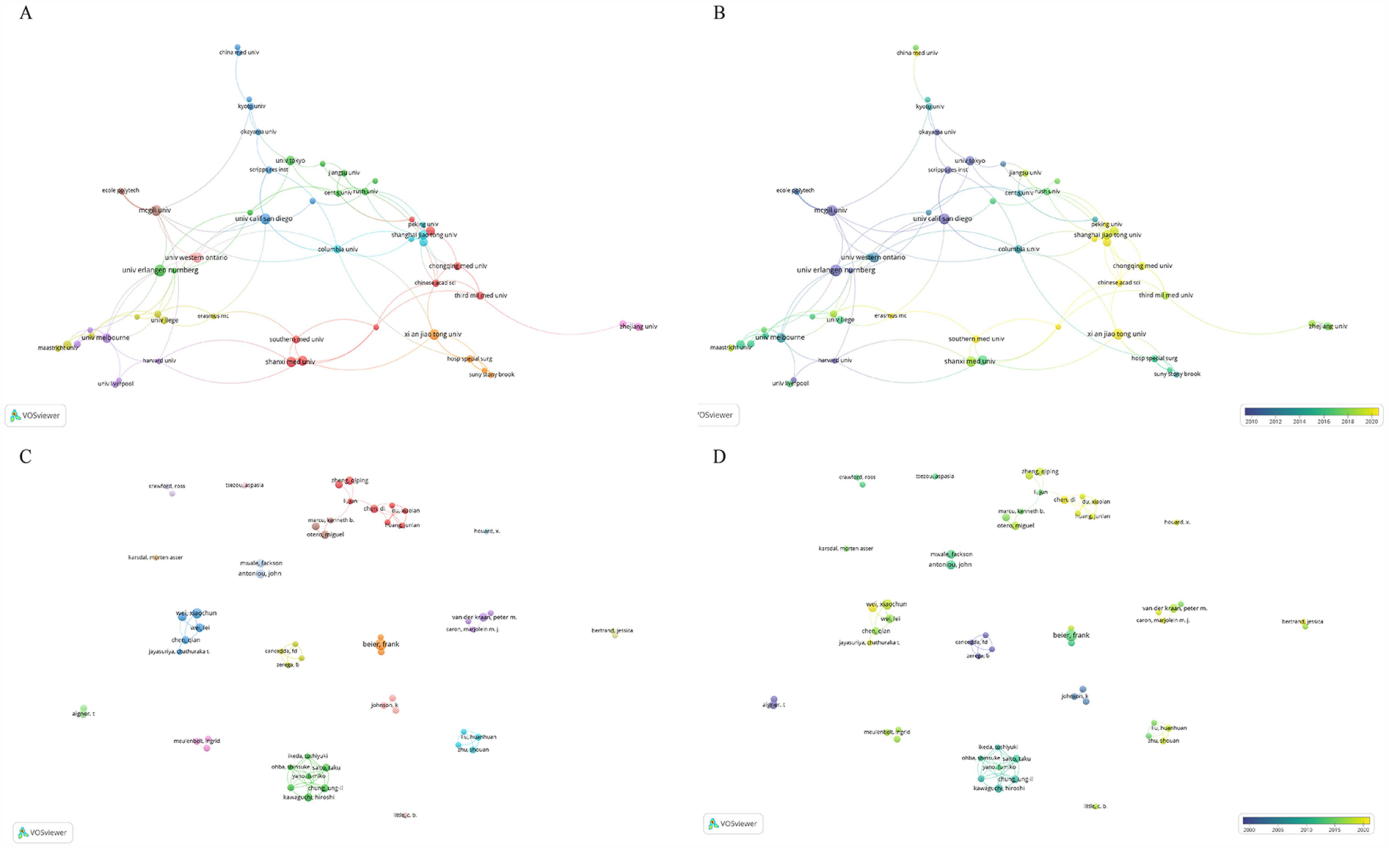
The analysis of institutions and authors. **(A,B)** The network map of institutions, the size of the nodes represented the publication number **(A)**. The color of the nodes represented the average appearing year **(B)**. **(C,D)** The network map of authors, the size of the nodes represented the publication number **(C)**. The color of the nodes represented the average appearing year **(D)**.

In terms of institutions, the most publications come from Xi'an Jiaotong University (*N* = 23), followed by the Sichuan University (*N* = 40). Table 4 presents the top ten institutions by publication volume. Figure 4A shows the co-authorship information between institutions, and Figure 4B presents the overlay visualization of these institutions. Table 5 displays the top ten authors by publication volume, and Table 6 shows the top ten authors by citation counts. Figure 4C illustrates the co-authorship analysis among authors in this field, while Figure 4D shows the overlay visualization. The author with the highest publication volume is BEIER F (*N* = 16), and the most cited author is KAWAGUCHI H (TC = 1,530). Based on publication volume, TC, and H-index, there is no absolutely core author in this field, indicating that the field is still developing and no single author has yet gained widespread influence.

**Table 4:** The top ten institutions with most publications.

Institution	Publications
Xi'an Jiaotong university	40
Sichuan university	33
University of California system	33
University of Erlangen Nuremberg	33
University of Western Ontario	29
Shanxi medical university	25
Shanghai Jiao Tong university	24
Sun Yat Sen university	23
Army medical university	22
University of Tokyo	20

**Table 5:** Top ten authors with most publications.

Author	Publications	Total Citations	H-index
Beier F	16	1,025	13
Van Der Kraan PM	12	1,001	10
Zhang Y	12	179	7
Terkeltaub R	10	1,371	10
Wei L	10	446	10
Antoniou J	10	248	8
Wang Q	10	220	8
Wei XC	10	310	8
Aigner T	9	1,081	9
Kawaguchi H	9	1,530	9

**Table 6:** Top ten authors with most citations.

Author	Total Citations	Publications	H-index
Kawaguchi H	9	1,530	9
Terkeltaub R	10	1,371	10
Nakamura K	6	1,321	6
Fosang AJ	6	1,244	6
Aigner T	9	1,081	9
Beier F	16	1,025	13
Werb Z	2	1,006	2
Van Der Kraan PM	12	1,001	10
Van Den Berg WB	5	892	5
Goldring MB	7	891	7

### 3.5 Analysis of keywords

**Figure 5:**
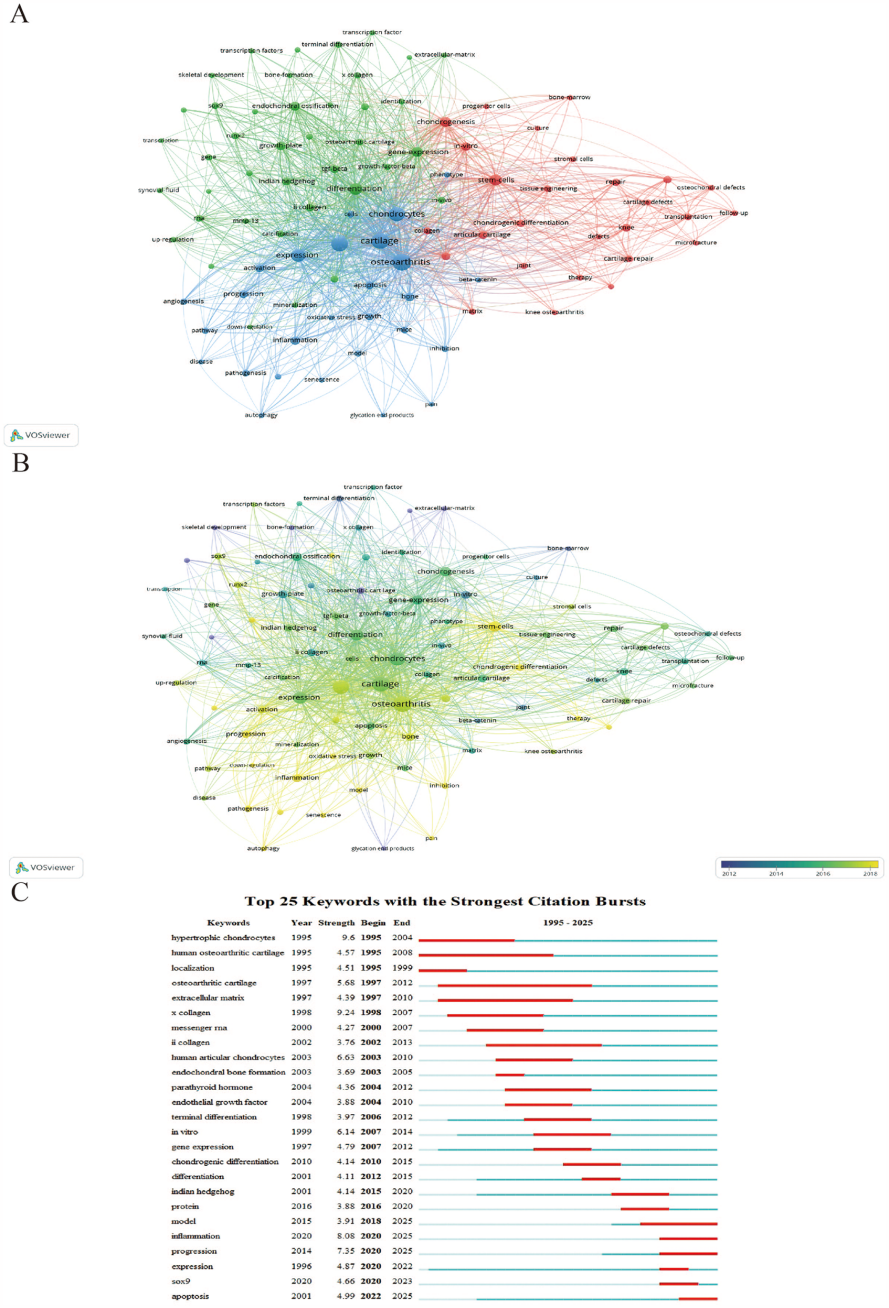
The analysis of keywords. **(A,B)** Network map of keywords, the size of the nodes represented the frequency **(A)** The color represented the average appearing year **(B)**. **(C)** Top 25 keywords with strongest citation bursts.

Among all keywords, the top five in terms of frequency are cartilage, osteoarthritis, expression, chondrocyte hypertrophy, and chondrocytes. Figure 5A presents the co-occurrence network of keywords generated through VOSviewer's clustering algorithm (resolution parameter = 1.0, minimum cluster size = 5), which objectively partitioned keywords into three distinct clusters. We define the red cluster as Cluster 1, the blue cluster as Cluster 2, and the green cluster as Cluster 3. Keywords in Cluster 1 are mostly related to clinical aspects, such as stem cells, cartilage repair, microfracture, tissue engineering, and transplantation. Cluster 2 mainly includes keywords related to pathological mechanisms involved in the disease, such as autophagy, aging, apoptosis, oxidative stress, inflammation. Cluster 3 encompasses pathological processes in the disease and related biomarkers, such as bone formation, mineralization, differentiation, transforming factors, growth factors, type X collagen, Runx2, MMP13, type II collagen, and TGF-beta. Figure 5B indicates that among all keywords, those that have appeared more recently are inflammation, autophagy, aging, oxidative stress, and stem cells. Additionally, we used CiteSpace to analyze the citation bursts of 25 keywords, as shown in Figure 5C.

## 4 Discussion

### 4.1 Summary of bibliometric characteristics

In this study, we conducted a comprehensive bibliometric analysis of the publications related to OA and chondrocyte hypertrophy. Based on the annual publication volume and our predictive model for future publications. Research in this field has grown steadily, attracting increasing attention. Regarding national contributions, China and the United States form the top tier, significantly leading other countries. However, it is noteworthy that although Chinese scholars have the highest publication volume, surpassing the United States, their TC is relatively low, with average citations lagging behind many countries. This could be attributed to the relatively late start of research by Chinese institutions and researchers, as shown in overlay visualizations of institutions and authors. It may also be due to the lower quality of research conducted by Chinese scholars. Notably, China's citation per publication (27.1) lags significantly behind the United States (64.9) and Germany (61.9), suggesting potential imbalances in research focus or collaborative depth that warrant strategic realignment.

We observed that apart from China, the main contributing countries in this field are all developed nations, likely benefiting from substantial healthcare expenditures and research funding support. However, we found that international collaboration in this field primarily exists among some of the high-contributing countries, suggesting that researchers should strengthen international cooperation in the future to promote the development of this field. It is noteworthy that there is no absolutely core author in this field. Among the high-impact authors, none lead in publication volume, citation counts, and H-index simultaneously. This phenomenon may be related to the rapid development of this field that no single author has yet formed a broad and profound influence. The collaborative network exhibits distinct characteristics of geographical proximity and disciplinary complementarity. Regional collaborative clusters between Chinese and Japanese, Chinese and American, as well as Chinese and Australian research institutions are primarily driven by geographical proximity, while cross-continental synergy relies on core hubs such as Harvard University and Shanghai Jiao Tong University for resource integration. Furthermore, basic research institutions and clinical centers establish synergistic partnerships through complementary expertise. For instance, the collaboration between Kyoto University and the Hospital for Special Surgery focuses on cartilage regeneration mechanisms, facilitating the rapid translation of fundamental discoveries into clinical applications.

In the analysis of high-impact journals, OSTEOARTHRITIS AND CARTILAGE is identified as the core journal in this field (*N* = 72, TC = 4,631, H-index = 37). Among the journals with high publication volumes, the journal with the highest impact factor is ANNALS OF THE RHEUMATIC DISEASES (IF = 27.4), indicating that research in this field has gained considerable attention and can be published in high-impact journals. These results show that research in this field is continuously growing globally, with findings increasingly being published in high-impact journals, indicating a promising development outlook and far-reaching influence.

### 4.2 Historical research trends and current hotspots

Based on keywords co-occurrence and overlay visualization analysis, we observed that the keywords in this field are mainly divided into three clusters. After comprehensive analysis, we identified these clusters as representing clinical research, pathological mechanisms, and pathological processes with molecular biological factors. Cluster 1 includes frequently occurring keywords such as stem cells, chondrogenesis, repair, microfracture, and transplantation. Microfracture surgery and cartilage transplantation are currently two important methods for treating cartilage injuries. However, post-surgery, the joint microenvironment may change due to factors such as growth factor secretion, inflammatory responses, and extracellular matrix remodeling, which can alter chondrocyte behavior and lead to chondrocyte hypertrophy (24).

Stem cells are considered crucial resources in regenerative medicine, with their self-renewal and multi-lineage differentiation potential. In recent years, significant progress has been made in the application of stem cells in cartilage repair. Stem cells enhance cartilage vitality and function through their ability to differentiate and secrete bioactive molecules (25). In 2020, Zhang et al. found that exosome-treated macrophages derived from bone marrow mesenchymal stem cells could maintain chondrogenesis and inhibit chondrocyte hypertrophy (26). Ali Mobasheri proposed that mesenchymal stem cells could address chondrocyte hypertrophy, a complication of autologous cartilage transplantation (27). Furthermore, some researchers have found promising results using mesenchymal stem cells combined with biomaterials in cartilage repair. Manjunatha S et al. discovered that Matrilin-3 hydrogel could significantly inhibit chondrocyte hypertrophy derived from adipose mesenchymal stem cells (28). In recent years, many authors have explored the field of tissue engineering. Tissue engineering materials, through special designs, can provide an ideal environment for chondrocytes (29). Combined with biocompatible materials, can mimic the cartilage extracellular matrix. This provides a three-dimensional space for cell proliferation and matrix synthesis, inhibiting chondrocyte hypertrophy (30). Some tissue engineering materials can also modulate chondrocyte behavior by applying specific mechanical stimuli, promoting the production of more specific matrices and preventing cartilage degradation and functional loss due to chondrocyte hypertrophy. These advancements present more promising solutions for clinical treatments. Tissue engineering and stem cell therapies show potential in OA treatment but face multiple challenges. Regarding therapeutic outcomes, multiple randomized controlled trials have demonstrated that mesenchymal stem cells can significantly alleviate pain, some studies have have shown potential cartilage protection or repair. The challenges primarily center on heterogeneity issues, controversies over dosage and sources, unclear mechanisms, uncertain long-term efficacy, and regulatory challenges (31).

Cluster 2 predominantly features pathological processes related to the phenotype of chondrocyte hypertrophy, such as senescence, inflammation, oxidative stress, autophagy, and apoptosis. Inflammatory responses play a crucial role in the pathogenesis of OA, particularly the inflammatory cytokines produced by chondrocytes and synovial cells, which can significantly alter the joint microenvironment (32). Chondrocytes exposed to this environment are may stimulated to undergo hypertrophy. In an inflammatory environment, mechanical stress and metabolic changes further complicate this process, leading to chondrocyte hypertrophy. This inflammatory milieu directly activates HIF-2α via NF-κB-p65 phosphorylation, triggering upregulation of MMP-13 and ADAMTS5 that synergistically drive hypertrophic differentiation (33). Understanding the role of inflammation in chondrocyte hypertrophy and its impact on overall joint health is crucial for designing effective therapeutic strategies (34). Studies have shown that the redox state of chondrocytes is vital in regulating chondrocyte differentiation and cartilage formation, ensuring appropriate responses to both endogenous and exogenous stimuli (35, 36).

In OA, oxidative stress can activate multiple cell signaling pathways, leading to the production and release of inflammatory cytokines. These cytokines further promote oxidative stress, creating a vicious cycle that contributes to chondrocyte hypertrophy and cartilage matrix degradation (37). Ying He et al. found that oxidative stress induces hypertrophic chondrocyte death by downregulating Smad2 protein expression, exacerbating cartilage damage (38). Ahmed MR et al. discovered that adipose-derived mesenchymal stem cells can reduce oxidative stress-induced chondrocyte damage and inhibit chondrocyte hypertrophy (39).

Apoptosis plays a crucial role in maintaining tissue homeostasis and removing harmful cells, while autophagy is a cellular cleanup mechanism through which cells degrade and recycle damaged organelles and proteins, maintaining internal environmental stability (40). Both processes are forms of programmed cell death. In chondrocytes, autophagy prevents the accumulation of abnormal proteins, preserving cell function. In OA, the function of autophagy may be compromised, leading to the inability to effectively clear intracellular damaged materials. When damaged materials accumulate within cells and are not effectively cleared by autophagy, apoptosis may be triggered (41). An imbalance in the regulation of apoptosis can result in the failure to timely remove hypertrophic chondrocytes, leading to their accumulation in cartilage tissue. This encroaches on the living space of healthy cells and leads to the secretion of inflammatory cytokines and degradative enzymes, further damaging the surrounding cartilage matrix (42).

Chondrocyte senescence is one of the key factors in cartilage degeneration in OA, involving permanent cell cycle arrest, accumulation of DNA damage, oxidative stress, and production of inflammatory cytokines (43). Senescent chondrocytes exhibit functional decline and release inflammatory cytokines, which further stimulate chondrocyte hypertrophy and exacerbate cartilage matrix degradation (10). They often fail to clear damaged proteins and organelles through autophagy, leading to the accumulation of intracellular toxins and accelerating hypertrophy (44). Senescent chondrocytes tend to die through apoptosis during their life cycle, and excessive apoptosis or the failure to clear damaged cells through apoptosis can negatively affect cartilage health (45). The interplay between chondrocyte autophagy, senescence, and apoptosis in OA determines the progression of cartilage degeneration (45, 46). Ye Lu et al. found that anti-Dlx5 delays OA progression by downregulating genes related to chondrocyte hypertrophy and apoptosis (47). Yu Zhang et al. used a thermosensitive hydrogel incorporating platelet-rich plasma to treat OA and found that this hydrogel increased local retention of exosomes, inhibiting chondrocyte apoptosis and hypertrophy (48). Lu Feng et al. discovered that MicroRNA miR-378 promotes OA by inhibiting chondrocyte autophagy and promoting chondrocyte hypertrophy, suggesting that regulating autophagy is an effective way to inhibit hypertrophy (49). Zhaoxun Chen et al. found that melatonin significantly inhibits oxidative stress-induced cartilage matrix degradation and chondrocyte apoptosis, while upregulating autophagy; *in vivo* experiments showed it alleviates cartilage ossification and chondrocyte hypertrophy (33). However, there are conflicting findings regarding the inhibition and upregulation of autophagy's effects on articular cartilage. Jiaji Yue et al. found that magnesium ions effectively inhibit chondrocyte hypertrophy-related genes Runx2, MMP13, and X collagen, and reduce the expression of autophagy protein LC3, thus protecting articular cartilage (50). Mao Xu et al. studied the effects of Sirt1 deletion in male mice with medial meniscal instability and observed its impact on OA development. They found that the loss of Sirt1 in cartilage accelerates OA progression through abnormal activation of the p53/p21-mediated senescence-associated secretory phenotype, hypertrophy, and apoptosis (51). Emőke Horváth et al. proposed that cellular senescence is the terminal functional stage of chondrocyte hypertrophy, with hypertrophy and senescence sharing many characteristics in chondrocytes, typically coexisting in a high-stress microenvironment. In OA pathology, hypertrophic chondrocytes gradually transform into senescent chondrocytes, regulated by signaling pathways such as Wnt, NOTCH, HIF-2α, and NF-kB, which collectively control inflammation and matrix degradation, thereby accelerating cartilage degeneration (52).

Cluster 3 primarily consists of bioactive factors and certain physiological processes. Type X collagen is a marker of chondrocyte hypertrophy; its overexpression typically signifies terminal differentiation of chondrocytes and is associated with cartilage matrix mineralization and degradation (53). MMP-13 is a matrix metalloproteinase whose expression is significantly increased in OA, particularly in hypertrophic and senescent chondrocytes. It can degrade type II collagen and other matrix components, thereby accelerating disease progression (54). Type II collagen is the main component of healthy cartilage, and its expression is significantly reduced in OA. Hypertrophic chondrocytes lose the ability to synthesize type II collagen, a crucial marker of cartilage health (54). Runx2 is a transcription factor closely related to the terminal differentiation and hypertrophy of chondrocytes. Its high expression can activate MMP-13 and other matrix components (55). SOX9 is an important transcription factor in chondrogenesis, regulating the expression of type II collagen and proteoglycans. In OA, certain inflammatory factors, such as IL-1β, can exacerbate cartilage matrix degradation by suppressing SOX9 expression (56). TGF-β plays a key regulatory role in the proliferation, differentiation, and matrix synthesis of chondrocytes. Its signaling pathway in OA shows dual effects: in some cases, TGF-β can promote cartilage repair and matrix synthesis; in others, it accelerates cartilage degeneration by promoting the expression of Runx2 and MMP-13 (57). These factors collectively regulate chondrocyte hypertrophy and senescence. Their abnormal expression leads to functional abnormalities in chondrocytes and matrix degradation, accelerating the development of OA. Understanding the interaction mechanisms of these factors is crucial for developing new therapeutic strategies for OA. By regulating the expression and function of these key factors, it may be possible to alleviate the hypertrophic phenotype of chondrocytes, protect the structure of cartilage tissue, and slow the progression of OA.

## 5 Conclusion

This study utilized bibliometric analysis methods to comprehensively analyze the bibliometric characteristics of publications in the intersecting field of OA and chondrocyte hypertrophy, elucidating the current status and development trends of this field. The field has been continuously developing in recent years, attracting increasing attention from researchers and making significant progress in understanding pathological mechanisms and potential therapeutic approaches. Key pathological mechanisms include inflammation, oxidative stress, apoptosis, autophagy, and senescence phenotypes, along with related bioactive factors during the OA process, which are focal points of research interest. In clinical treatment, researchers are mainly focused on stem cells and tissue engineering. Notably, our study revealed the parallel research trends in “stem cell therapy” “tissue engineering” highlighting their synergistic potential in modulating chondrocyte hypertrophy and providing a roadmap for future interdisciplinary collaboration. Future research should continue to focus on the regulatory mechanisms of these key phenotypes and factors, strengthen international cooperation, and improve research quality to provide more effective strategies and treatment of OA.

## 6 Limitation

Although this study reveals the research status and development trends in the intersecting field of OA and chondrocyte hypertrophy through systematic bibliometric analysis, there are several limitations to consider. Firstly, our data are entirely sourced from the Web of Science Core Collection (WOSCC). While it includes a large amount of high-quality academic publications, it does not encompass publications from other databases such as PubMed and Scopus, which may lead to biased results. Inherent biases in bibliometric analysis—such as citation bias (overrepresentation of highly cited articles), self-citation practices inflating specific authors' impact, and language bias (underrepresentation of non-English studies)—may skew the interpretation of research trends. The analysis methods used in this study rely on statistical and bibliometric techniques, although they provide an overview of research trends and hotspots. But there were limitations in interpreting specific research content and quality. Additionally, bibliometric analysis often has a time lag effect, where newly published high-impact papers have not yet received sufficient citations and may be underestimated in the analysis. Furthermore, the inherent limitations of keyword-based clustering algorithms might oversimplify interdisciplinary research themes, potentially overlooking nuanced connections between emerging subfields. Future research should consider the comprehensive utilization of multiple databases and the combination of quantitative analysis with qualitative assessment to further enhance the comprehensiveness and accuracy of bibliometric analysis.

## Data Availability

Publicly available datasets were analyzed in this study. This data can be found here: Web of Science Core Collection.
